# The association between the romantic relationships of parents and offspring depressive symptoms: Mediating effects of offspring communication patterns and romantic relationships

**DOI:** 10.3389/fpsyg.2022.897380

**Published:** 2022-08-11

**Authors:** Na Li, Yi-Meng Zhang, Na-Na Xiong, Qi-Qing Sun, Ying Qian, Hong-Qiang Sun

**Affiliations:** Peking University Sixth Hospital, Peking University Institute of Mental Health, NHC Key Laboratory of Mental Health, National Clinical Research Center for Mental Disorders (Peking University Sixth Hospital), Beijing, China

**Keywords:** romantic relationship, communication patterns, depressive symptoms, mediating role, parental relationship

## Abstract

This study investigated a conceptual model by testing how parental romantic relationships influenced the depressive symptoms of grown-up children and whether the constructive communication patterns of grown-up children and romantic relationships played mediation effects within it. A total of 421 Chinese participants were enrolled in the study. The level of depressive symptoms, romantic relationship satisfaction and closeness, couple communication patterns, and parental romantic relationships were measured *via* self-report questionnaires. According to the results, the structural equation modeling analysis verified that the severity of participants’ depressive symptoms was negatively associated with the parental romantic relationship and that the association was mediated by participants’ constructive communication patterns and their own romantic relationships. Furthermore, compared with nondepressed participants, depressed participants were less satisfied with their parental romantic relationships, exhibited fewer constructive communication patterns, and were more distant and unsatisfied with current romantic relationships.

## Introduction

Depression is a leading cause of disability and years of productive life lost worldwide (McCarron et al., 2021). It was ranked second among the top 25 leading causes of years lived with disability (YLDs) and accounted for the largest proportion of mental disorder disability-adjusted life-years (DALYs) in 2019 (37.3% [32.3–43.0]; GBD, 2022). The global economic cost associated with depression is expected to double by 2030 (McCarron et al., 2021). It is important to improve strategies to prevent and intervene in depression. However, only approximately half of patients with depression receive adequate treatment. While medication often relieves depressive symptoms, it does not address the accompanying social dysfunctions, such as problems with relationships, family, and work. The presence of interpersonal problems, psychosocial stressors, or comorbid personality disorders is generally considered to suggest the need for nonpharmacological interventions (McCarron et al., 2021). Exploring the association between romantic relationships and depressive symptoms is expected to provide evidence supporting the development of nonpharmacological intervention strategies for depression.

Studies have found that romantic relationships between parents are inversely associated with increased depressive symptoms in children (Clavarino et al., 2011). A longitudinal study of 153 new parents found that parental conflict in the first year of life was associated with internalized outcomes (such as depressive symptoms) at age 6 and that a 1-unit increase in parental use of angry conflict resolution styles was associated with a 4.46-unit increase in child internalizing problems (Craft et al., 2021). Researchers have attempted to address issues with parental romantic relationships to alleviate depressive symptoms in their children. In many cases, however, the influence of parental romantic relationships on offspring depression does not emerge until their offspring grow up and have their own romantic relationships (White et al., 2021). Clinicians often find that some depressive children complain about their parents being responsible for their depressive symptoms. A study of 93 families by Bodner et al. found that depressed adolescents experienced harsher and more conflictual interactions with both of their parents compared to nondepressed adolescents (Bodner et al., 2018). Unfortunately, solidified parental relationships are difficult to change. It is important to investigate mediating variables in the impact of parental romantic relationships on the depressive symptoms of offspring. Future studies could explore whether the depressive symptoms of grown-up children might be prevented or relieved through interventions involving these mediating variables. There have been studies focusing on investigating parent–child interactions as mediating roles (Sugawara et al., 2002; Tian et al., 2022). No research was conducted from the perspective of offspring communication patterns and romantic relationships. It has been reported that communication patterns and romantic relationships might transfer from the original family to the offspring’s current family (Wang et al., 2010; Miller et al., 2013). Therefore, the current study decided to develop a chain model with a view to clarifying the impact of parental romantic relationships on offspring depressive symptoms.

## Development of the model

### Parental romantic relationships and depressive symptoms in offspring

Romantic relationships are multidimensional, including facets such as satisfaction, stability, and closeness (Fletcher et al., 2000; Cao et al., 2017). Previous studies have indicated that parental romantic relationships are related to the risk of offspring depression. Gilman et al. performed a follow-up study with 1,104 participants who had experienced family disruption during childhood and found an association between family disruption, particularly divorce, and adult children’s depression (Gilman et al., 2003). They also found that parental conflict was independently related to a high lifetime risk of depression in adults. However, the exact underlying mechanisms of how parental romantic relationships affect depression in grown-up children remain unclear. Therefore, we propose the following hypothesis:

*H1-1*: The level of parental romantic relationships is negatively associated with depressive symptoms in offspring; the lower the level of parental romantic relationships from the perspective of children, the higher the score of depressive symptoms in offspring.

### The potential mediation effects of the current romantic relationship and communication pattern

Romantic relationships were reported to be transferred from parents to their children (Wang et al., 2010, 2014; Miller et al., 2013; Cao et al., 2017). In other words, couples often feel distant and unsatisfied in their own romantic relationship when their parental romantic relationship is far away and discontented. A distant and unsatisfactory romantic relationship was reported to be a powerful predictor of depression in couples by The Marital Discord Model of Depression (MDMD; Hamburg, 1991; Maroufizadeh et al., 2018). Closeness and satisfaction with romantic relationships were found to be important influencing factors of depression. Miller et al. tested the MDMD in 391 couples and found that satisfaction with romantic relationships significantly predicted their own depressive symptoms (Miller et al., 2013). Kim et al. and Chung et al. both showed that romantic relationship closeness was negatively associated with depressive symptoms (Chung, 2016; Kim and Kim Hee, 2018). Thus, a lack of satisfaction and closeness in romantic relationships might be associated with depression. Therefore, we hypothesize that the parental romantic relationship might influence depressive symptoms in an individual through their current romantic relationship.

*H1-2*: The romantic relationship of grown-up children plays a mediating role in the impact of parental romantic relationships and depression. Grown-up children who have a lower-level assessment of parental romantic relationships will have less closeness and satisfaction in their own romantic relationships with a higher score of depressive symptoms.

Couples who experienced satisfactory parental romantic relationships showed more constructive communication patterns than couples with divorced parents (Sanders et al., 1999). Based on a long-term follow-up of 213 participants, Cui et al. found that interparental aggression was significantly correlated with the offspring’s target to spouse/partner verbal aggression (r = 0.39, *p* < 0.05; Cui et al., 2010). Evidence also showed that communication patterns between couples could predict the severity of depressive symptoms over time (Laurent et al., 2009). Another study included 63 American couples and found that the communication pattern of female demand/male withdrawal was positively associated with depressive level (r_male partner_ = 0.27, *p* < 0.05; r_female partner_ = 0.28, *p* < 0.05; Li and Johnson, 2018). Barry et al. also found that depressive symptoms were positively associated with disengaged communication (β = 0.21, *p* < 0.01; Barry et al., 2019). Hence, the parental romantic relationship might influence the depressive symptoms of individuals by affecting their communication patterns.

*H1-3*: Communication patterns also play a mediating role in the impact of parental romantic relationships and depression. Grown-up children who have a lower-level assessment of their parental romantic relationship will have less constructive and more nonconstructive communication patterns with a higher score of depressive symptoms.

In addition, previous work supported a positive association between communication patterns and romantic relationships of grown-up children. For example, Olson has indicated that communication patterns play a key role in promoting the development of closeness and adaptability in a romantic relationship (Olson et al., 1979). Geiss et al. found that nonconstructive communication patterns (such as accusation, avoidance, defense, etc.) were the most destructive in romantic relationships (Geiss and O'Leary, 1981). In contrast, the research on 431 newlyweds by Lavner et al. showed that constructive communication patterns were significantly associated with higher romantic relationship satisfaction (beta_wife_ = 0.16, beta_husband_ = 0.20) and predicted relationship satisfaction after 9 months (Lavner et al., 2016). Liu et al. also found that constructive communication patterns were positively associated with the adaptability of romantic relationships (beta = 0.28; Liu et al., 2014). However, the exact pathways among these influences and outcomes remain unclear. Taken together, these pieces of evidence lead us to propose our assumption that:

*H1-4*: Communication patterns and current romantic relationships sequentially mediate the relationship between parental romantic relationships and the depressive symptoms of an individual.

Last, previous studies did not explore the differences between depressive and nondepressive individuals regarding the parental romantic relationship in their original family and communication patterns or romantic relationship in their current family. Li et al. conducted a study including 148 Chinese outpatients with marriage problems from a mental health hospital and 400 normal married participants. The results showed that compared with the control group, the depressed group scored higher on male demand/female withdrawal and female demand/male withdrawal communication patterns but scored lower on constructive communication (*p* < 0.05; Li et al., 2018b). In Western countries, there were similar findings that the depressed group showed a significantly worse quality of marital relationship compared to the healthy control group, and the clinical couples reported less constructive communication (Lemmens et al., 2007; Kronmüller et al., 2011). Previous studies also indicated that poor parental marital quality may cause emotional unpleasantness in family relations and threaten offspring emotional problems (Tian et al., 2022). Thus, our study aims to explore such differences, and our hypothesis is that:

*H2*: Compared with nondepressive individuals, depressive individuals have a less satisfying parental romantic relationship in their original family and show less constructive communication patterns and fewer close and satisfying romantic relationships in their current family.

## Materials and methods

### Participants and procedure

This investigation carried out a cross-sectional survey from February 2020 to June 2021 in China. A web-based questionnaire approach was adopted considering the epidemic prevention and control measures at that time, and a convenience sampling method was employed. We recruited participants from the community and from Peking University Sixth Hospital. The inclusion criteria were as follows: age 16–65 years; experience with romantic relationships; and ability to provide fully informed consent online. All the participants were required to complete online self-report measures, and they were offered free couples counseling by the researchers. A total of 446 participants completed the self-report questionnaire. Of the returned questionnaires, nonserious answers and missing values were excluded; thus, data from 25 participants were excluded from the final analysis. A total of 421 valid questionnaires were obtained, for an effective rate of 94.39%. The data included in the final analysis were from 260 females (61.8%) and 161 males (38.2%). The participants were 36.62 ± 10.09 years old. This research was approved by the Ethical Committee of the Peking University Sixth Hospital.

### Measurements

#### Depressive symptoms

The Patient Health Questionnaire-9 (PHQ-9) is a self-assessment screening for depression. Nine symptoms of depression are measured on a four-point scale as follows: 0 (rarely or none), 1 (a few days), 2 (more than half of the days in 2 weeks), and 3 (almost every day). Subjects with scores above 4 on the PHQ-9 are considered to exhibit depressive symptoms. We adopted the Chinese version of the PHQ-9. It has been verified to have good reliability and validity in previous studies (Chen et al., 2013; Sun et al., 2020).

#### Romantic relationship satisfaction

Romantic relationship satisfaction was assessed by the six-item Quality Marriage Index (QMI; Norton, 1983). The scale has six questions: the first five are seven-point scales ranging from 1 (extremely disagree) to 7 (extremely agree), and the last item asks couples to indicate how happy they are in their relationship, all things considered, on a 10-point scale ranging from 1 (very unsatisfied) to 10 (very satisfied). Higher scores indicate higher levels of satisfaction. The Cronbach’s alpha coefficient in our study was 0.968.

#### Romantic relationship closeness

Romantic relationship closeness was evaluated by the Inclusion of Other Scale (IOS; Aron et al., 1992). The IOS is a single-item pictorial measure that contains seven pairs of overlapping circles, which represent the relationship between the two people on a 7-point scale ranging from 1 (not close at all) to 7 (very close). The greater the overlap between the circles, the greater the inclusion of the partner in the self.

#### Parental romantic relationship

The parental romantic relationship was assessed by one well-designed question for the grown-up children. “In your eyes, what is your assessment of your parents’ romantic relationship?” It had a visual analog scale ranging from 0 to 10. Zero means in your eyes, your parents are totally unsatisfied with their relationship. Ten means that in your eyes, they feel fully happy, intimate, and contented with their relationship. Systemic family therapy held the theory that an individual’s own view of the fact plays a more important role than the fact itself (Von Schlippe et al., 2018). According to this theory, the own ideas of grown-up children about the assessment of parental romantic relationships might play a more important role in the association between parental romantic relationships and the mental conditions of grown-up children than other types of assessments of their parental romantic relationships.

#### Couple communication patterns

The communication patterns questionnaire (CPQ) was used to assess the communication patterns of participants and their partners (Christensen and Shenk, 1991). It included three parts with 16 total items. The communication patterns were evaluated from three aspects: when the problem occurred when the problem was discussed and after the problem was discussed. Each item assesses the partners’ perception of how likely a certain type of behavior (e.g., both members avoid discussing the problem) occurs when faced with a relationship problem, from 1 (very unlikely) to 9 (very likely). The higher the score was, the higher the frequency with which couples exhibited a given communication pattern. The questionnaire has three subscales and explores four factors: constructive communication, mutual avoidance, self-demand/partner withdraw, and partner-demand/self-withdraw. Their definitions are detailed in Table 1 (Christensen and Shenk, 1991; Heavey et al., 1996). The Chinese version of the CPQ has been widely used, and the Cronbach’s alpha coefficient was 0.73 (Zhang et al., 2009).

**Table 1:** Definition of the six communication patterns.

Communication pattern	Definition
Constructive communication	The sum of three items assessing constructive communication behaviors minus the sum of four items assessing destructive communication behaviors.
Mutual avoidance	The sum of three items that assess couples’ mutual avoidance, mutual withdrawal, and mutual withholding
Self-demand/partner-withdraw	Three items measure when an individual presses their partner to discuss a problem and then makes demands, criticizes, and nags them, while the partner tries to avoid discussion, withdraw, or is silent.
Partner-demand/self-withdraw	Three items measure when an individual’s partner presses them to discuss a problem and then makes demands, criticizes, and nags them, while the individual tries to avoid discussion, withdraw, or is silent.

### Data analysis

The data were analyzed using SPSS version 24.0 (IBM) and Mplus version 7.4 (Muthén and Muthén, 2015). The normal distribution of continuous variables was assessed using the Shapiro–Wilk test of normality: age, educational background, family monthly income, parental romantic relationship, current communication patterns, and current relationships were nonnormally distributed. The Mann–Whitney *U*-test was used to compare the depressed and nondepressed groups. A Chi-square test was conducted to compare sex ratios between the groups. Among the demographic traits, there were significant differences in age and sex, but there were no significant differences in educational background or family monthly income between the groups. We used partial correlation analysis to examine associations among parental romantic relationships, current communication patterns, current relationships, and current depressive symptoms, controlling for age and sex. Then, confirmatory factor analysis was performed to determine whether the observed variables that comprised each latent variable in the structural equation model were properly constructed. Structural equation modeling (SEM) was used to verify the model and calculate the direct and indirect path coefficients of factors influencing the model. The 95% confidence intervals were calculated by bootstrapping with 1,000 replications. We adopted the suggested cutoff criteria for a good fit as follows: comparative fit index (CFI) ≥ 0.90, the Tucker–Lewis index (TLI) ≥ 0.90, and root mean square error of approximation (RMSEA) ≤ 0.08 (Hu and Bentler, 1999; Byrne, 2010).

## Results

### Characteristics of depressed and nondepressed participants

A total of 66.7% of participants reported depressive symptoms according to the Patient Health Questionnaire-9 (PHQ-9). Parental romantic relationships and current romantic relationships showed significantly lower scores in the depressed group than in the nondepressed group (z = −4.685, *p* < 0.001). Regarding couple communication patterns, the constructive communication score of the depressive group was significantly lower than that of the nondepressed group (z = −3.774, *p* < 0.001); however, no significant difference between the two groups was found in nonconstructive communication patterns. There was no significant difference in family monthly income (χ^2^ = 3.597, *p* = 0.731) between the groups with and without depressive symptoms (Table 2).

**Table 2:** Sample characteristics of depressed and nondepressed participants.

Variable	Participants with depressive symptoms (*n* = 281)	Participants without depressive symptoms (*n* = 140)	Z**/**χ^2^	Value of *p*
Age	34.00	40.00	−3.918	<0.001
Sex			12.278	<0.001
Male	91(32.4)	70(50.0)		
Female	190(67.6)	70(50.0)		
Education level			3.597	0.731
High school or below	43(15.3)	17(12.1)		
Junior college and bachelor	153(54.5)	84(60.0)		
Master or above	85(30.2)	39(27.8)		
Family monthly income (RMB)			11.637	0.040
<3,000	12(4.3)	3(2.1)		
3,000 ~ 5,000	19(6.8)	11(7.9)		
5,000 ~ 10,000	64(22.8)	36(25.7)		
10,000 ~ 20,000	84(29.9)	25(17.9)		
20,000 ~ 30,000	40(14.2)	33(23.6)		
>30,000	62(22.1)	32(22.9)		
Parental romantic relationship	8.00	7.00	−4.685	<0.001
**Current communication patterns**				
Constructive communication	12.00	6.00	−3.774	<0.001
Mutual avoidance	9.00	10.00	−1.403	0.161
Self-demand/partner-withdraw	13.00	13.00	−0.010	0.992
Partner-demand/self-withdraw	11.00	12.00	−0.033	0.974
**Current romantic relationship**				
Closeness	6.00	5.00	−4.805	<0.001
Satisfaction	43.00	36.00	−5.662	<0.001
Current depressive symptoms	1.50	10.00	−16.752	<0.001

### Correlation analysis among the parental romantic relationship, depressive symptoms, communication patterns, and current romantic relationship

Correlation analysis was conducted among parental romantic relationships and depressive symptoms, communication patterns, and romantic relationships (Table 3). We found that parental romantic relationship quality was positively associated with the current romantic relationship (closeness: r = 0.21, *p* < 0.001, satisfaction: r = 0.21, *p* < 0.001) and negatively associated with depressive symptoms (r = −0.22, *p* < 0.001) in all participants. The constructive communication pattern was positively associated with parental romantic relationship quality (r = 0.16, *p* < 0.001) and current romantic relationships (closeness: r = 0.63, *p* < 0.001, satisfaction: r = 0.61, *p* < 0.001) and negatively associated with other nonconstructive communication pattern factors and depressive symptoms (r = −0.26, *p* < 0.001). Depressive symptoms were negatively associated with current romantic relationships (closeness: r = −0.28, *p* < 0.001, satisfaction: r = −0.36, *p* < 0.001).

**Table 3:** Correlations among variables.

	1	2	3	4	5	6	7
1. Parental romantic relationship	1.00						
2. Offspring’s constructive communication	0.16^**^	1.00					
3. Offspring’s mutual avoidance	−0.03	−0.50^***^	1.00				
4. Offspring’s self-demand/partner-withdraw	0.01	−0.37^***^	0.50^***^	1.00			
5. Offspring’s partner-demand/self-withdraw	−0.03	−0.26^***^	0.47^***^	0.52^***^	1.00		
6. Offspring’s closeness of current romantic relationship	0.21^***^	0.63^***^	−0.21^***^	−0.10^*^	0.06	1.00	
7. Offspring’s satisfaction in current romantic relationship	0.21^***^	0.61^***^	−0.21^***^	−0.10	0.04	0.83^***^	1.00
8. Offspring’s depressive symptoms	−0.22^***^	−0.26^***^	0.11^*^	0.07	0.05	−0.^28^^***^	−0.36^***^

* Correlation is significant at the 0.05 level (2-tailed).

** Correlation is significant at the 0.01 level (2-tailed).

*** Correlation is significant at the 0.001 level (2-tailed).

The associations between the quality of parental romantic relationships and mutual avoidance communication patterns were not significant. However, mutual avoidance was negatively correlated with current romantic relationship quality and positively correlated with depressive symptoms (*r*_romantic relationship closeness_ = −0.21, *p* < 0.001; *r*_romantic relationship satisfaction_ = −0.21, *p* < 0.001; *r*_depressive symptoms_ = 0.11, *p* < 0.05).

The associations between the parental romantic relationship and self-demand/partner-withdraw communication patterns were not significant. The self-demand/partner-withdraw communication pattern was negatively correlated with current romantic relationship quality (self-demand/partner-withdraw: *r*_romantic relationship closeness_ = −0.10, *p* < 0.05; *r*_romantic relationship satisfaction_ = −0.14, *p* < 0.01) but was not significantly associated with depressive symptoms.

### SEM of the parental romantic relationship, depressive symptoms, communication patterns, and romantic relationship

**Figure 1:**
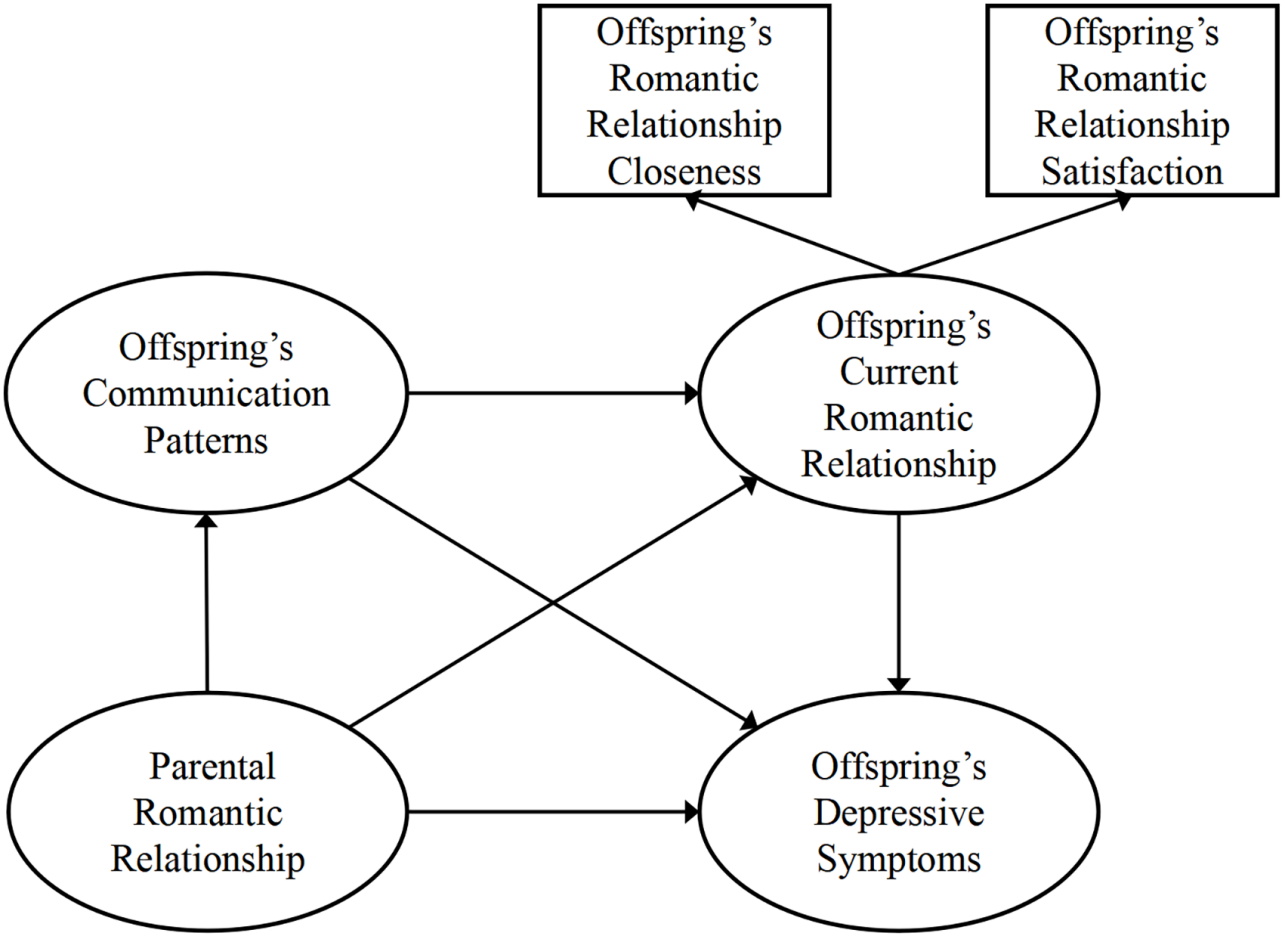
Conceptual model of the study.

**Figure 2:**
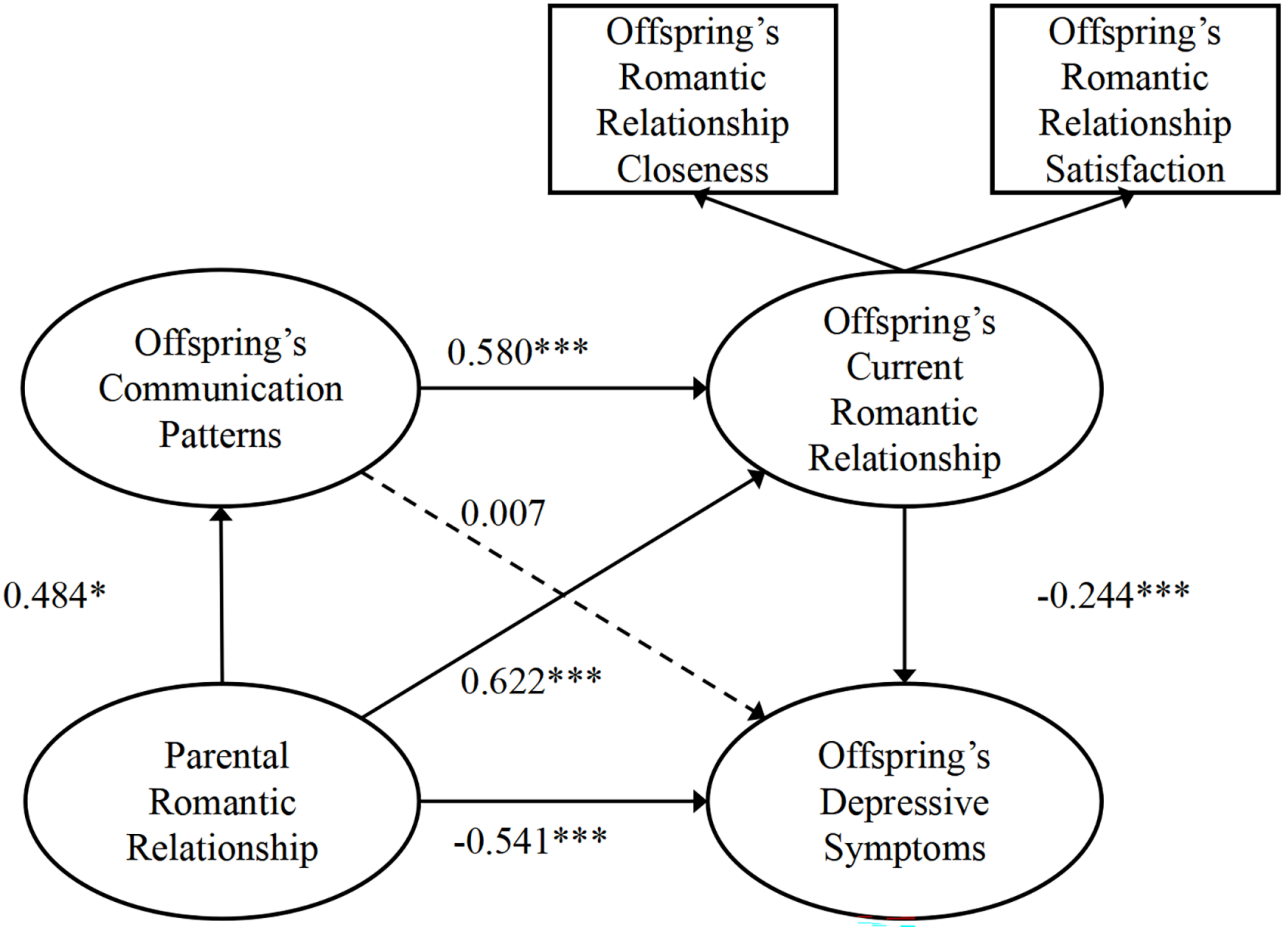
The associations among the parental romantic relationship and the offspring’s constructive communication patterns, current romantic relationship, and depressive symptoms ^*^p < 0.05; ^**^p < 0.01; ^***^p < 0.001.

Based on our review of the literature, we constructed a theoretical model incorporating parental romantic relationships and communication patterns, romantic relationships, and depressive symptoms, as shown in Figure 1. We speculated that parental romantic relationships might affect depressive symptoms through communication patterns and current romantic relationships (Figure 1). Thus, we conducted SEM to test our proposed theoretical model; the model indices are presented in Table 4. Each communication pattern was examined separately. When fitting the data, we found that all the communication pattern models showed acceptable model indices, and the comparative fit indexes (CFI) and Tucker–Lewis indexes (TLI) were all > 0.9. Then, we tested the mediating role of each communication pattern and found that only the constructive communication pattern model showed a significant chain mediating effect (Table 5). In this model, we found a significant total effect of the parental romantic relationship on depressive symptoms: *β* = −0.757, 95% CI: [−0.980, −0.532], *p* < 0.05. The direct effect of the parental romantic relationship on depressive symptoms was significant, *β* = −0.541, 95% CI: [−0.744, −0.300], *p* < 0.05, which indicated a partially mediated model. The indirect effect through the current romantic relationship was also significant: *β* = −0.152, 95% CI: [−0.254, −0.077], *p* < 0.05, as was the indirect effect through constructive communication and the current romantic relationship: *β* = −0.068, 95% CI: [−0.148, −0.024], *p* < 0.05. However, the indirect effect through constructive communication alone was not significant: *β* = 0.004, 95% CI: [−0.031, 0.052], *p* > 0.05 (Table 5). We present each effect value among parental romantic relationships, constructive communication patterns, current romantic relationships, and depressive symptoms of participants in Figure 2.

**Table 4:** The model indices.

Index	*χ* ^2^	*df*	CFI	TLI	RMSEA	Value of *p*
1. Constructive communication	12.69	2	0.987	0.935	0.113	<0.01
2. Mutual avoidance	4.93	2	0.995	0.976	0.059	0.085
3. Self-demand/partner-withdraw	3.62	2	0.997	0.987	0.044	0.164
4. Partner-demand/self-withdraw	3.42	2	0.998	0.988	0.041	0.181

CFI, comparative fit index; TLI, Tucker–Lewis index; RMSEA, root mean square error of approximation.

**Table 5:** Results of the chain mediating effects.

Model	Constructive communication		Mutual avoidance communication		Self-demand/partner-withdraw		Partner-demand/self-withdraw		*β*	95% CI	*β*	95% CI	*β*	95% CI	*β*	95% CI
Direct effect	−0.541^***^	−0.744, −0.300	−0.543^***^	−0.751, −0.319	−0.558^***^	−0.760, −0.330	−0.558^***^	−0.760, −0.333
Parental romantic relationship → offspring’s depressive symptoms								
	−0.757^***^	−0.980, −0.532	−0.757^***^	−0.980, −0.532	−0.757^***^	−0.980, −0.532	−0.757^***^	−0.980, −0.532
Total indirect effect	−0.217^**^	−0.329, −0.120	−0.215^***^	−0.328, −0.118	−0.199^**^	−0.305, −0.104	−0.200^**^	−0.306, −0.105
Parental romantic relationship → offspring’s communication pattern → offspring’s current romantic relationship → offspring’s depressive symptoms								
	−0.068^*^	−0.148, −0.024	0.007	−0.011, 0.007	0.002	−0.003, 0.017	−0.002	−0.015, 0.001
Parental romantic relationship → offspring’s communication pattern → offspring’s depressive symptoms								
	0.004	−0.031, 0.052	−0.002	−0.027, 0.006	0.002	−0.003, 0.028	0.001	−0.004, 0.024
Parental romantic relationship → offspring’s current romantic relationship → offspring’s depressive symptoms								
	−0.152^**^	−0.254, −0.077	−0.220^***^	−0.331, −0.130	−0.203^**^	−0.318, −0.115	−0.199^**^	−0.305, −0.107

* *p* < 0.05.

** *p* < 0.01.

*** *p* < 0.001.

## Discussion

Our study supported a chain mediation model in which the current communication patterns and romantic relationships of grown-up children mediated the association between the parental romantic relationship and current depressive symptoms. This model was raised for the first time. We also found differences in current couple communication patterns and romantic relationships between depressed and nondepressed participants.

According to the current results, the correlation between a parental romantic relationship and depressive symptoms of grown-up children was higher in the chain mediation model (*β* = −0.757) than their direct correlation (*β* = −0.541). This means that the constructive communication patterns of grown-up children and romantic relationships played a great sequential mediation role in the impact of parental romantic relationships on offspring depression. In other words, the more constructive communication of individuals, the more closeness and satisfaction of the romantic relationship quality, and the greater the negative impact of parental romantic relationships on offspring depression. These findings are consistent with previous studies. Sanders et al. found that couples who experienced satisfied parental romantic relationships showed more constructive communication patterns than couples with divorced parents (Sanders et al., 1999). Constructive communication patterns positively predicted future relationship quality (Heene et al., 2005). Furthermore, Miller et al. found that the romantic relationship satisfaction of people with depression was negatively associated with their own depressive symptoms (Miller et al., 2013). Overall, constructive communication patterns might play a mediating role in the impact of parental romantic relationships on the current romantic relationship of offspring, thereby preventing depressive symptoms. Such findings could be explained by family system theory and social learning theory (Kronmüller et al., 2011; Miller et al., 2013). Family system theory posits that the romantic relationship and communication patterns exhibited by parents are transferred across generations. Social learning theory suggests that children learn from their parents (Li et al., 2018a). Therefore, a romantic relationship between parents can serve as a blueprint for a romantic relationship in offspring (Miller et al., 2013; Cao et al., 2017). Therefore, constructive communication patterns might be transferred from parents to their children, which can improve future romantic relationships; in such a manner, constructive communication patterns might prevent the emergence of depressive symptoms. The chain mediation model in our study provides meaningful evidence for a resource-oriented view of preventing depression. Most previous studies have focused on nonconstructive communication patterns and attempted to link symptoms of mental illness with these communication patterns (Heaven et al., 2006; Jarnecke et al., 2016; Holley et al., 2018). However, our study did not find a mediating effect of nonconstructive communication patterns on the association between parental romantic relationships and offspring depressive symptoms. These interesting findings might guide future studies to pay more attention to resource-oriented variables, such as constructive communication patterns, in the prevention or treatment of depression. However, these findings remain controversial. Baucom et al. did not find any direct relationship between depressive symptoms and communication behaviors (Baucom et al., 2007). Due to the relatively small sample size of this study, it is important for future studies to recruit more participants to evaluate our chain mediation model.

Regarding nonconstructive communication patterns, our study found that mutual avoidance could mediate current romantic relationships and depression. This finding is consistent with the findings of Zhao (2008), Jarnecke et al. (2016), and Holley et al. (2018). However, we also obtained some controversial findings. Baucom et al. performed a follow-up study on 134 seriously and chronically distressed married couples and found that destructive communication patterns were not significantly associated with 2- and 5-year romantic relationship satisfaction (Baucom et al., 2015). Additionally, Iverson et al. did not find an association between reduced nonconstructive communication patterns and improvements in romantic relationship satisfaction after treatment (Iverson and Baucom, 1990). This inconsistency might be explained by variation in nonconstructive communication patterns. The current study treated mutual avoidance and demand/withdrawal patterns as different types of nonconstructive communication. We found that mutual avoidance, rather than demand/withdrawal communication patterns, was the mediating variable between current romantic relationships and depression. However, the studies by Baucom et al. and Iverson et al. combined mutual avoidance and demand/withdraw communication patterns during analysis. Therefore, future studies should separately analyze mutual avoidance and demand/withdraw communication patterns and further investigate mutual avoidance communication patterns. However, we found no mediating effect of mutual avoidance on the association between parental romantic relationships and current romantic relationships of offspring. Other nonconstructive communication patterns (such as mutual blame) could play a mediating role in the impact of the association between the parental romantic relationship and the current romantic relationship. Therefore, in-depth research on nonconstructive communication patterns is urgently needed.

Compared to nondepressed participants, depressed participants exhibited more unsatisfactory parental romantic relationship quality, less constructive communication, more nonconstructive communication, and more distant and unsatisfactory current romantic relationship quality. These findings are consistent with previous studies. Johnson et al. indicated that spouses with more frequent depressive episodes are less likely to actively communicate with their partners (Johnson and Jacob, 2000). Systemic theorists have proposed that nonconstructive communication patterns within couples might maintain individual symptoms, such as depression (Christensen and Shenk, 1991). Therefore, people with depressive symptoms might have bidirectional correlations between parental romantic relationships, current communication patterns, and current romantic relationships (Li et al., 2018a).

In addition, the unique features of Chinese social contexts may have special theoretical value to examine the above issue (Tian et al., 2022). Many studies from China and Western countries have indicated that the original family has a great influence on offspring. Chinese people from traditional families attach more importance to family values. Our findings should be interpreted with caution when generalizing them to other cultural samples.

There are some implications for future directions and applications. First, grown-up children with depressive symptoms might be deeply troubled by unsatisfied parental romantic relationships. Some of them complained about their parents, but the parental romantic pattern was difficult to change. This study provided evidence that interventions that target current communication patterns and romantic relationships of grown-up children might help mitigate the negative impact of parental romantic relationships on their mental health and subsequently alleviate depressive symptoms. Second, this study indicated that for grown-up children, constructive communication behavior could not only mitigate the negative impact of parental romantic relationships on their own mental health but also be good examples for their own children when they become parents. Therefore, they could learn to use more constructive communication patterns and to build close and satisfying romantic relationships. Last, the results of this study provide a direction for future studies to develop interventions for the enhancement of constructive communication between husbands and wives to solve problems of the original family to prevent and treat depression.

Our study had the following limitations. First, measurements of communication patterns are subjective. Future research could adopt more objective measurements and analyze the communication patterns and parental romantic relationships in reality; for example, multiple observers could evaluate video recordings of couples communicating. Second, there was only one question about parental romantic relationships of participants. In the future, more structured questionnaires could be used to assess different domains of parental romantic relationships, such as closeness, satisfaction, and gender. Third, couple communication patterns might be related to many factors, such as child neglect and domestic violence. More potential confounding factors should be considered in future studies to make the study design more rigorous. Finally, although we investigated mediating factors in the association between parental romantic relationships and offspring depressive symptoms, cross-sectional research designs cannot establish causal relationships. Therefore, longitudinal designs or interventions should be applied to explore the underlying mechanisms.

## Data availability statement

The original contributions presented in the study are included in the article/Supplementary material, further inquiries can be directed to the corresponding authors.

## Ethics statement

The studies involving human participants were reviewed and approved by the Ethical Committee of the Peking University Sixth Hospital. The ethics committee waived the requirement of written informed consent for participation.

## Author contributions

YQ, NL, and H-QS developed the research question and study design. NL and Y-MZ oversaw the data analysis. NL, Y-MZ, and YQ collected the data. NL and Y-MZ contributed to the data interpretation and writing of the article. N-NX, Q-QS, YQ, and H-QS revised the article. All authors contributed to the article and approved the submitted version.

## Funding

This work was supported in part by grants from the National Key R&D Program of China (2021YFF0306500) and the Beijing Municipal Science and Technology Commission (Z191100006619047).

## Conflict of interest

The authors declare that the research was conducted in the absence of any commercial or financial relationships that could be construed as a potential conflict of interest.

## Publisher’s note

All claims expressed in this article are solely those of the authors and do not necessarily represent those of their affiliated organizations, or those of the publisher, the editors and the reviewers. Any product that may be evaluated in this article, or claim that may be made by its manufacturer, is not guaranteed or endorsed by the publisher.
